# IPRStats: visualization of the functional potential of an InterProScan run

**DOI:** 10.1186/1471-2105-11-S12-S13

**Published:** 2010-12-21

**Authors:** Ryan J Kelly, David E Vincent, Iddo Friedberg

**Affiliations:** 1 Department of Computer Science and Software Engineering, Miami University, Oxford OH 45056, USA; 2 Department of Microbiology Pearson Hall, 700 E. High St. Miami University, Oxford OH 45056, USA

## Abstract

**Background:**

InterPro is a collection of protein signatures for the classification and automated annotation of proteins. Interproscan is a software tool that scans protein sequences against Interpro member databases using a variety of profile-based, hidden markov model and positional specific score matrix methods. It not only combines a set of analysis tools, but also performs data look-up from various sources, as well as some redundancy removal. Interproscan is robust and scalable, able to perform on any machine from a netbook to a large cluster. However, when performing whole-genome or metagenome analysis, there is a need for a fast statistical visualization of the results to have good initial grasp on the functional potential of the sequences in the analyzed data set. This is especially important when analyzing and comparing metagenomic or metaproteomic data-sets.

**Results:**

IPRStats is a tool for the visualization of Interproscan results. Interproscan results are parsed from the Interproscan XML or EBIXML file into an SQLite or MySQL database. The results for each signature database scan are read and displayed as pie-charts or bar charts as summary statistics. A table is also provided, where each entry is a signature (e.g. a Pfam entry) accompanied by one or more Gene Ontology terms, if Interproscan was run using the Gene Ontology option.

**Conclusions:**

We present an platform-independent, open source licensed tool that is useful for Interproscan users who wish to view the summary of their results in a rapid and concise fashion.

## Background

Function analysis of protein sequences is one of the primary challenges in the post-genomic era [1]. As sequencing technology becomes cheaper, we are becoming inundated with sequencing data, which requires annotation and interpretation. Computationally annotating gene and protein function is a difficult problem for several reasons, and is best solved if attacked by several different strategies. First, in many cases we know only certain aspects of a protein's function. We may predict that a protein is a protease, but not know which protein or proteins it degrades. Conversely, we may know that a protein plays a role in a specific pathway, but not its molecular function. At the same time, this knowledge does not preclude the protein's participation in other pathways, of which we know nothing about. Many function prediction algorithms use homology-based transfer, the rationale being that functional similarity can be inferred from sequence similarity. However, homology-based transfer algorithms require, first and foremost, a comprehensive, accurately annotated and up-to-date reference sequence database, but no single database can boast all three traits at 100% [2]. This is true for pairwise sequence alignment algorithms, simple sequence-motif algorithms, as well as for the more complex profile hidden Markov models (pHMM) [3] and position specific sequence similarity based algorithms [4]. It is therefore almost obligatory to use several function annotation programs to functionally annotate proteins. The rationale is that by using more than one algorithm to functionally annotate a protein, we overcome the lack of sensitivity that may result from using only one program. Furthermore, a consensus method can help weed out false positives. For those reasons, Interproscan [5] is a popular function annotation program. Interproscan compares query protein sequences against Interpro -a repository of collected and annotated protein signatures- member databases using a variety of motif, pHMMs and positional specific score matrix methods. This ability to compile results from different sequence signature methods makes Interproscan the software of choice for protein function annotation. Since Interproscan also scales up to work on cluster computers, it can handle large amounts of data. However, when producing large amounts of data, there is a need to provide a good visualization of the results to make them tractable. With the advent of fast genome sequencing, and with the increasing number of studies involving metagenomics and metaproteomics, it is has become customary to talk about the *functional potential* of a microbiome or a genome (e.g. [6]). Functional potential is defined as the biochemical and physiological functions that the analyzed group of genes or proteins may perform. For example, a comparative analysis of the gut microbiome of obese and lean mice has shown that the constituent bacteria of obese mice have a higher count of enzymes that break down complex carbohydrates [7]. In a study of marine microbial samples, Gianoulis *et al.* have found correlations between the genomic potential of the samples and the environmental conditions. In water samples that were nutrient-poor (as indicated by chlorophyll content) there were more pathways that had to do with amino acid synthesis than in samples that were in nutrient-rich water. This is because bacteria in nutrient poor water are selected for the ability to synthesize amino acids that are otherwise unavailable [8]. Other examples include the functional potential of *Haloferax volcanii DS2* which has been shown to be greatly increased with a variety of membrane transporters, with respect to other archaeal species in its clade [9]. Whereas the functional potential of *Mycoplasma,* a genus of parasitic bacteria has been shown to be lacking in many basic metabolic functions, as *Mycoplasma* ingest nutrients from their hosts [10]. While Interproscan provides the data constituting the functional potential, IPRStats lets the user visualize it. Some software packages produce a visualization of the functional potential of a large scale analysis. Those include the online resources RAST [11] and MG-RAST [12], IMG/M [13], and RAMMCAP [14]. However, these resources require that the initial functional annotation be performed using the tools provided by them, which sets limitations on choices the annotator may want to make. There is therefore a need for software to provide an overview of the functional potential of a genome or a metagenome annotated by Interproscan. Here we present such software, which we call IPRStats: Interproscan Statistics. It uses the output of Interproscan as its input, and quickly produces charts and tables enabling a visualization of the functional potential of the sequences analyzed.

## Implementation

### General

**Figure 1:**
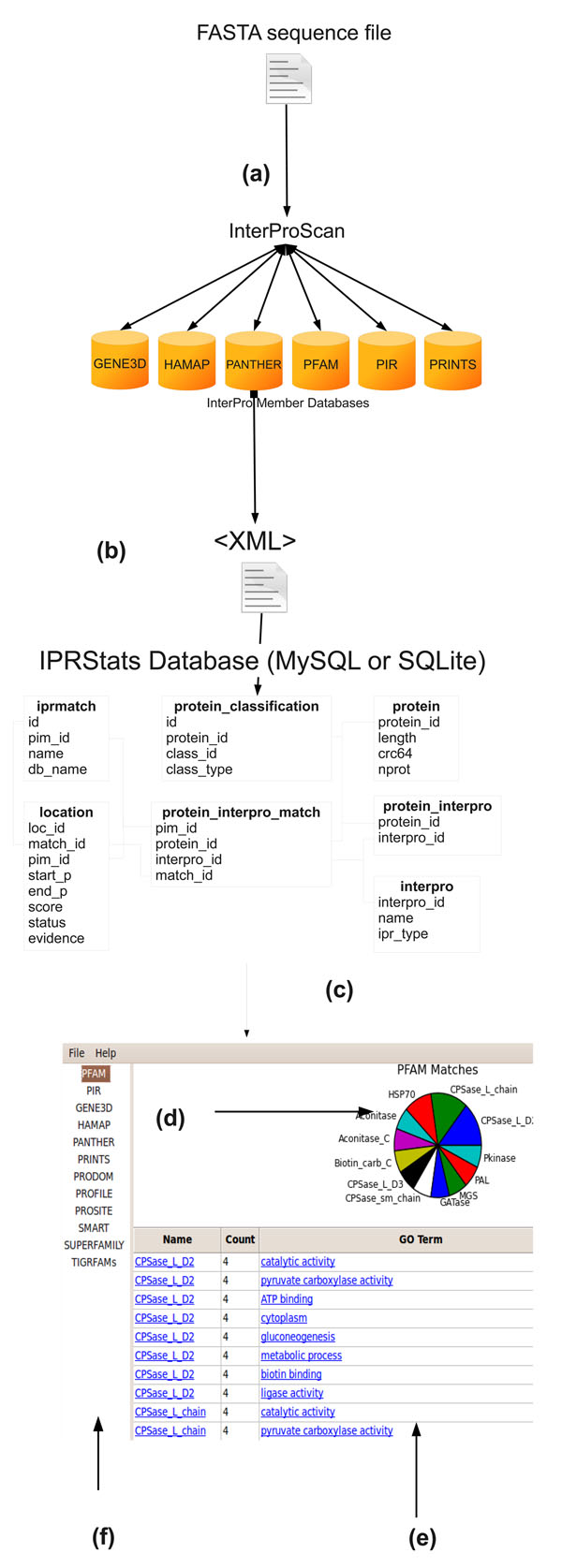
Flowchart of information in IPRStats (a) Protein sequence information as a single FASTA file submitted to Interproscan (one or more proteins) (b) Interproscan XML output imported into IPRStats SQL database (c) Display of sequence signature statistics (d) graphic display (e) table display (f) Toggle between results from different InterPro member databases

Figure 1 describes the flow of information in IPRStats. The output of an Interproscan run is stored in XML format. The XML file is parsed into a 7-table SQLite [15] or a MySQL [16] database. The tables follow the data structure outlined by the Interproscan XML schema and the tables can persist for queries by software other than IPRStats. After reading the tables, IPRStats displays the information alphanumerically and graphically. The tabs in the sidebar of the main program screen toggle between the display of results for each sequence signature program called by Interproscan. The results display includes a graphic chart (Figure 1d) and a table (Figure 1e). The chart is either a pie chart or a bar chart, which shows the count of different sequence signatures from the relevant program in the analyzed sequence population. Chart drawing is implemented using either Google Chart Tools [17], or matplotlib [18]. Google Chart Tools is a web-based API that dynamically generates charts using a URL string, so when drawing using Google Chart Tools an active Internet connection is required. Alternatively, matplotlib may be used: matplotlib is a Python-based clone of MatLab, which can be used for chart graphics as well, and does not require an Internet connection.

The table (bottom) includes the same information as the chart, and also Gene Ontology (GO) annotations, if relevant. GO [19] is a controlled vocabulary of functional terms which is a widely used standard for describing gene and gene product attributes. By default, the table includes all relevant GO numeric IDs produced by Interproscan, each linked to a descriptive web page at the European Bioinformatics Institute (EBI). The numeric GO IDs can be translated to more legible terms, but that requires an optional connection to a local or a remote GO database. If using a remote connection, the query time can slow the initial data loading time considerably.

### Data export

IPRStats data can be exported either in HTML for view using a browser, or in Microsoft^®^ Excel^TM^. HTML exports include all graphics, so those can be viewed with a browser. Excel^TM^ exports can be further processed using standard electronic spreadsheet tools. Additionally, an IPRStats work session may be saved as a binary file for later import.

### Web server version of IPRStats

IPRStats can also be installed as a web server. The installation follows simple guidelines for an Apache server based installation. The user can upload Interproscan-generated XML files. This option is recommended for smaller amounts of data. Currently there is one installation at http://wan1.mbi.muohio.edu/iprstats

### Availability

IPRStats is written in Python, with a graphic user interface (GUI) based on wxWidgets, a cross-platform toolkit for graphic user interfaces. Relying on platform-independent fully open source infrastructure ensures that we maximize portability of IPRStats. Currently IPRStats has been tested on Windows XP/7, Max OS X 10.6 and Ubuntu GNU/Linux 9.10 and 10.04. IPRStats is downloadable from Github at http://github.com/devrkel/IPRStats. Packages for Windows, Mac and Linux (.deb) are available at http://github.com/devrkel/IPRStats/downloads

## Conclusions

IPRStats is a lightweight, platform-independent open-source licensed software package for viewing and initial interpretation of results from Interproscan. We welcome further development by the community. Please contact the corresponding author for details.

## List of Abbreviations

EBI: Eupropean Bioinformatics Institute; GO: Gene Ontology; IMG/M: Integrated Microbial Genome/Metagenomics; pHMM: Profile Hidden Markov Model; RAST: Rapid Anotation using Subsystem Technology; MG-RAST: Metagenome RAST; RAMMCAP: Rapid analysis of Multiple Metagenomes with Clustering and Annotation Pipeline; SQL: Structured Query Language; XML: Extensible Markup Language;

## Competing Interests

All authors declare they have no competing interests.

## Authors contributions

IF conceived of the idea, planned the database schema, and wrote the XML-to-database code. DEV and RJK wrote the web version, and the graphics exporter. RJK wrote the standalone version, distribution packaging, and documentation. All three authors were involved in software design. IF and RJK wrote the manuscript.
